# Factors Affecting the Baseline and Post-Treatment Scores on the Hopkins Verbal Learning Test-Revised Japanese Version before and after Whole-Brain Radiation Therapy

**DOI:** 10.3390/ijms17111834

**Published:** 2016-11-03

**Authors:** Hirotake Saito, Kensuke Tanaka, Ayae Kanemoto, Toshimichi Nakano, Eisuke Abe, Hidefumi Aoyama

**Affiliations:** 1 Department of Radiology and Radiation Oncology, Niigata University Graduate School of Medical and Dental Sciences, 1-757 Asahimachi-dori, Chuo-ku, Niigata 951-8510, Japan; ktanaka@med.niigata-u.ac.jp (K.T.); prague2657@yahoo.co.jp (T.N.); eabe@med.niigata-u.ac.jp (E.A.); 2 Department of Radiation Oncology, Niigata Cancer Center Hospital, 2-15-3 Kawagishi-cho, Chuo-ku, Niigata 951-8566, Japan; ayae-kanemoto@umin.ac.jp

**Keywords:** brain metastasis, whole-brain radiation therapy, Hopkins Verbal Learning Test-Revised, neurocognitive function

## Abstract

Our objectives were to (1) investigate the feasibility of the use of the Japanese version of the Hopkins Verbal Learning Test-Revised (HVLT-R); (2) identify the clinical factors influencing the HVLT-R scores of patients undergoing whole-brain radiation therapy (WBRT); and (3) compare the neurocognitive function (NCF) after WBRT in different dose fractionation schedules. We administered the HVLT-R (Japanese version) before (baseline) and at four and eight months after WBRT in 45 patients who received either therapeutic (35Gy-in-14, *n* = 16; 30Gy-in-10, *n* = 18) or prophylactic (25Gy-in-10, *n* = 11) WBRT. Sixteen patients dropped out before the eight-month examination, due mostly to death from cancer. The Karnofsky Performance Status (KPS) 80–100 group had significantly higher baseline total recall (TR) scores (*p* = 0.0053), delayed recall (DR) scores (*p* = 0.012), and delayed recognition (DRecog) scores (*p* = 0.0078). The patients aged ≤65 years also had significantly higher TR scores (*p* = 0.030) and DRecog scores (*p* = 0.031). The patients who underwent two examinations (worse-prognosis group) had significantly decreased DR scores four months after WBRT compared to the baseline (*p* = 0.0073), and they were significantly more likely to have declined individual TR scores (*p* = 0.0017) and DR scores (*p* = 0.035) at four months. The eight-month HVLT-R scores did not significantly decline regardless of the WBRT dose fractionation. The baseline NCF was determined by age and KPS, and the early decline in NCF is characteristic of the worse-prognosis group.

## 1. Introduction

In the last decade, the importance of both whole-brain radiation therapy (WBRT) and stereotactic radiosurgery (SRS) in the management of brain metastasis (BM) has been highlighted again with the latest improvement in prognostic stratification. Sperduto et al. published the results of a secondary analysis of the Radiation Therapy Oncology Group (RTOG) 9508 trial comparing WBRT + SRS with WBRT alone in the management of 1–3 BMs [1]. In that analysis, non-small cell lung cancer (NSCLC) was the dominant primary tumor. The patients with favorable prognoses showed improved survival when treated with WBRT + SRS compared to those treated with WBRT alone (*p* = 0.05). In their secondary analysis of the JROSG 99-1 trial, Aoyama et al. post-stratified the NSCLC patients. They reported that the patients with BM from NSCLC in the favorable-prognosis group had longer survival when treated with WBRT + SRS compared to those treated with SRS alone (*p* = 0.04) [2]. The difference in overall survival was not observed in the worse-prognosis group (*p* = 0.86). For the patients with a good systemic condition and a small number of systemic metastases, improved intracranial tumor control could lead to prolonged survival.

Although both WBRT and SRS are indispensable in the management of BM, two trials demonstrated a negative impact of WBRT on the patients’ neurocognitive function (NCF) at 3–4 months after the treatment, and these results set the trend toward the SRS-alone strategy [3,4]. It is of note that the Hopkins Verbal Learning Test-Revised (HVLT-R) was used in both of those studies, and this test is considered to have adequate properties to assess the NCF after brain irradiation; the English version of the HVLT-R has been increasingly used in trials dealing with brain metastasis and other brain tumors [5].

The objectives of the present study were to (1) investigate the feasibility of the use of the Japanese version of the HVLT-R; (2) identify the clinical factors influencing the HVLT-R (Japanese version) scores of patients who have undergone WBRT; and (3) compare the patients’ NCF after WBRT in different dose fractionation schedules.

## 2. Results

### 2.1. Patient Characteristics

The characteristics of the 45 patients are listed in Table 1. The 25-Gy group was comprised of only the LD-SCLC patients who were assigned to prophylactic WBRT (*n* = 11), and they had no intracranial metastases at the beginning of the WBRT. Sixteen patients (36%) underwent HVLT-R at four months after WBRT but did not undergo the eight-month examination. The remaining 29 patients (64%) underwent the eight-month examination. The most common reason for the discontinuation of HVLT-R was death from cancer (*n* = 8). The proportion of patients who did not undergo the eight-month examination was significantly higher in the Karnofsky Performance Status (KPS) ≤ 70 group compared to the KPS ≥ 80 group (Table 2, *p* = 0.014 by Fisher’s exact test).

### 2.2. Factors Affecting the Baseline HVLT-R Scores

Table 3 shows the association between the baseline scores of the subdomains TR, DR, DRecog of the HVLT-R, and the clinical factors. The patients with KPS ≥ 80 had significantly higher TR scores (*p* = 0.0053 by Mann-Whitney *U*-test), higher DR scores (*p* = 0.012), and higher DRecog scores (*p* = 0.0078) compared to the patients with KPS ≤ 70. The patients aged ≤65 years also had significantly higher TR scores (*p* = 0.030) and higher DRecog scores (*p* = 0.031) compared to the patients aged ≥66 years, and they tended to have higher DR scores (*p* = 0.080). There were no significant differences in any HVLT-R subdomain scores among the WBRT dose groups, or among the different types of the cranial lesion.

### 2.3. HVLT-R Raw Scores of the Patients Who Underwent Two Examinations (Baseline and Four Months) (Worse-Prognosis Group, n = 16)

**Figure 1:**
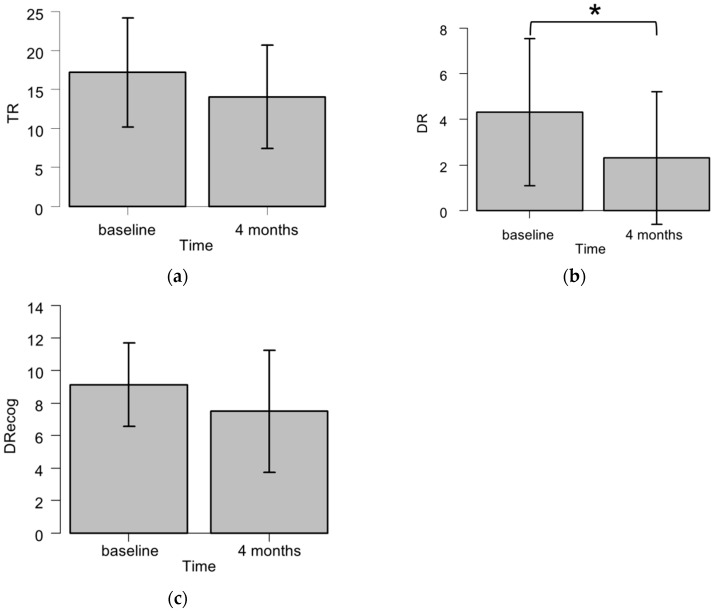
The Hopkins Verbal Learning Test-Revised (HVLT-R) total recall (TR) (**a**); delayed recall (DR) (**b**); and delayed recognition (DRecog) (**c**) scores of the patients who underwent the examination at the baseline and four months after WBRT (*n* = 16). The raw scores are indicated as mean ± SD. * *p* < 0.01 by Wilcoxon signed rank test.

Figure 1 summarizes the HVLT-R subdomain scores in the patients who did not undergo the eight-month examination. The DR scores were significantly decreased four months after WBRT compared to the baseline (*p* = 0.0073 by Wilcoxon signed rank test). The TR scores tended to decline four months after WBRT (*p* = 0.057), but this change was not observed in the DRecog scores (*p* = 0.15).

### 2.4. HVLT-R Raw Scores of the Patients Who Underwent Three Examinations (Baseline, Four, and Eight Months) (Better-Prognosis Group, n = 29)

**Figure 2:**
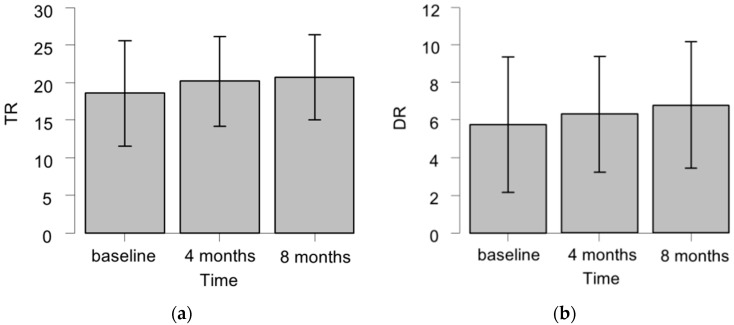

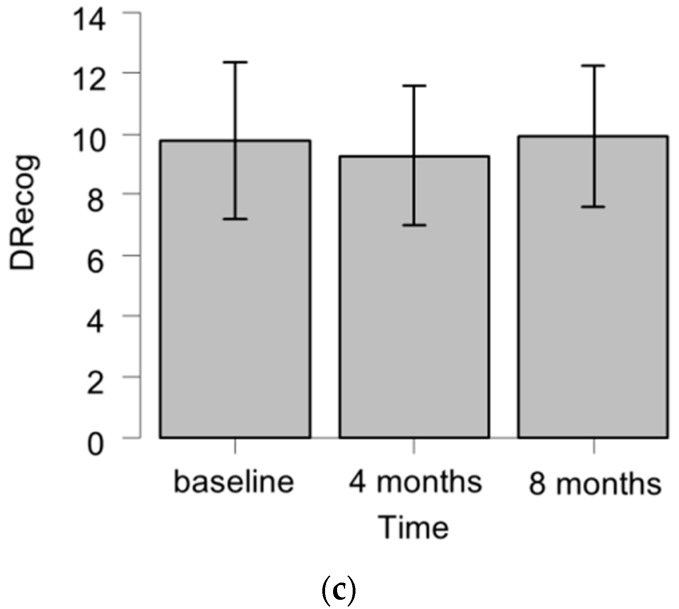
The HVLT-R TR (**a**); DR (**b**); and DRecog (**c**) scores of the patients who underwent the examination at the baseline, four months, and eight months after WBRT (*n* = 29). The raw scores are indicated as mean ± SD.

Figure 2 summarizes the HVLT-R subdomain scores in the patients who also underwent the eight-month examination. There were no significant changes over time in the TR scores (*p* = 0.24 by Friedman test), DR scores (*p* = 0.14), or DRecog scores (*p* = 0.13).

Table 4 shows the HVLT-R scores of the patients whose total number of examinations was three (baseline, four, and eight months) by WBRT dose fractionation. In the 25-Gy group, the four-month TR scores were significantly higher than the baseline scores (Bonferroni-adjusted *p* = 0.045). To test the hypothesis that the patients who underwent 25-Gy WBRT and three examinations had worse baseline TR scores compared to the other WBRT dose groups, we compared the baseline TR scores of each WBRT dose group by Kruskal-Wallis test. There were no significant differences in the baseline TR scores among the WBRT dose groups (*p* = 0.40). The eight-month HVLT-R scores were not significantly declined in any subdomain regardless of the WBRT dose fractionation.

### 2.5. Factors Associated with a Significant Decline in Individual HVLT-R Scores at Four Months

Table 5 shows the proportion of significant decline in four-month individual HVLT-R scores and clinical factors. The patients with KPS ≤ 70 were significantly more likely to have declined TR scores at four months compared to the patients with KPS ≥ 80 (Fisher’s exact test, *p* = 0.013). Similarly, the patients who underwent two examinations (worse-prognosis group; baseline and four months) were significantly more likely to have declined TR scores (*p* = 0.0017) and DR scores (*p* = 0.035) compared to the patients who underwent three examinations (better-prognosis group; baseline, four, and eight months).

### 2.6. Factors Associated with a Significant Decline in Individual HVLT-R Scores at Eight Months

Table 6 shows the proportion of significant decline in eight-month individual HVLT-R scores and clinical factors. The patients aged ≥66 years tended to have declined DR scores at eight months compared to the patients aged ≤65 years (*p* = 0.060).

## 3. Discussion

This is the first clinical study in which the Japanese version of the HVLT-R was used to investigate the serial changes in NCF after brain irradiation. By applying this test, we were able to assess subtle differences or changes before and after brain irradiation, which might not be detected by the Mini-Mental State Examination (MMSE).

At the baseline, the patients with either older age or lower KPS exhibited lower HVLT-R scores. A similar association was observed with the MMSE. Aoyama et al. reported that BM patients aged ≥66 years or those with KPS ≤ 80 had lower baseline MMSE scores than their counterparts [6]. Kurita et al. used the MMSE to analyze the NCF of 1915 cancer patients who underwent treatment with opioids [7], and they reported that older age and lower KPS were independently associated with MMSE scores <27.

Although it has been used widely, a weak point of the MMSE is that it is designed to evaluate global cognitive function rather than learning and memory. It also has a ceiling effect [8]. Sun et al. published the results of their secondary analysis of the RTOG 0214 trial evaluating the effect of prophylactic WBRT for advanced NSCLC [9]: the rate of decline in individual TR scores of the HVLT was significantly higher in the prophylactic WBRT arm at 3, 6, and 12 months, whereas the rate of decline in individual MMSE scores was higher in the intervention arm only at three months. The ability of the HVLT-R to detect subtle changes in learning and memory supports its routine use in trials evaluating cranial radiation therapy for BM.

One of the interesting findings of the present study is that a significant decline in the score at four months was observed only in the patients who underwent the examination twice (worse-prognosis group; baseline and four months) but not in the patients who underwent the examination three times (better-prognosis group; baseline, four, and eight months). Sixteen (36%) of our patients underwent the four-month HVLT-R but dropped out before the eight-month examination. Their DR scores declined significantly compared to the baseline, and they were also significantly more likely to have declined individual TR and DR scores at four months compared to the patients who completed the four-month examination. The reason for dropping out by eight months was mostly death from cancer or worsened general condition. The KPS ≤ 70 at the baseline was a risk factor for discontinuation of the HVLT-R and declined individual TR scores at four months. The KPS is regarded as a prognostic factor of several primary cancer sites, and it is reasonable that the patients with a low KPS have worse survival and cannot undergo repeated HVLT-R examinations.

Cognitive deterioration determined by the HVLT-R at three or four months was employed as a primary endpoint in two randomized clinical trials (RCTs) comparing SRS alone and SRS + WBRT for patients with one to three brain metastases [3,4]. In a recent publication from North America, Brown et al. reported that there was less cognitive deterioration at three months after SRS alone (40/63, 63.5%) than when SRS was combined with WBRT (44/48, 91.7%). In a smaller RCT from the MD Anderson Cancer Center, Chang et al. reported that patients who were randomly assigned to receive SRS + WBRT were significantly more likely to show a decline in learning and memory function (mean posterior probability of decline 52%) at four months than patients assigned to receive SRS alone (mean posterior probability of decline 24%). It should be noted that the former (Brown et al.) study recruited 213 patients (SRS alone, *n* = 111, SRS + WBRT, *n* = 102); however, only 57% (63/111) of the patients in the SRS-alone group and 47% (48/102) of the patients in the SRS + WBRT group underwent HVLT-R at three months. Similarly, in the Chang et al. study, only 67% (20/30) of the patients in the SRS-alone group and 39% (11/28) of the patients in the SRS + WBRT group underwent the HVLT-R at four months. The results of the present study suggest that the four-month decline in NCF is characteristic of the lower-KPS group, and this early decline must be distinguished from the true long-term toxicities that affect the favorable-prognosis group. This finding is supported by the RTOG 0933 trial evaluating hippocampus-avoiding WBRT; Gondi et al. reported that the HVLT-R DR scores of the patients who had died by six months declined significantly over time [10]. This decline was not observed in the patients who were alive at six months.

Regarding the association between the schedule of WBRT and the HVLT-R score among the 29 patients who underwent both the four- and eight-month tests in the present study, neither the total dose nor the fractionation of WBRT affected the four- and eight-month HVLT-R scores. Wolfson et al. conducted the RTOG 0212 trial comparing three different prophylactic WBRT dose fractionations in LD-SCLC [11]. They used the HVLT, the Controlled Oral Word Association Test (COWAT), and the Trail Making Test (TMT) parts A and B. Chronic neurotoxicity was defined as deterioration in at least one test without the development of brain metastases at 12 months. In a logistic regression analysis, chronic neurotoxicity was associated with the WBRT dose fractionation of 36 Gy in 18 fractions and older age. It seems difficult to assess the relationship between the WBRT dose fractionation and the incidence of 12-month decline in the NCF of BM patients, because the median survival time for all BM patients is approximately eight months [12]. The late adverse effects of therapeutic WBRT for BM will be of clinical importance for long-term survivors.

Our study has several limitations. First, the study population was heterogeneous because we compared the NCF of patients who had undergone different WBRT fractionation schedules; Second, the small sample size made it difficult to elucidate possible confounders with a multivariate analysis; Finally, the degree of radiation-induced NCF deterioration might be overestimated, because it was difficult to eliminate the effect of the regrowth of intracranial tumors.

## 4. Materials and Methods

### 4.1. Patient Population

We enrolled the patients who were assigned to therapeutic WBRT for the treatment of BM, skull metastasis, dural metastasis, leptomeningeal dissemination of cancer, or central nervous system (CNS) involvement of non-Hodgkin lymphoma (NHL) from April 2012 to May 2014 at Niigata University Hospital and Niigata Cancer Center Hospital. We also included patients with limited-disease small cell lung cancer (LD-SCLC) who were assigned to prophylactic WBRT. Patients with a neurological deficit, such as a consciousness disorder, hemiparesis, visual defect, or aphasia, that could disturb the neuropsychological examinations were excluded. The dose fractionation of therapeutic WBRT was 35 Gy in 14 fractions over 3 weeks at Niigata University Hospital, and 30 Gy in 10 fractions over 2 weeks at Niigata Cancer Center Hospital. The fractionation of prophylactic WBRT was 25 Gy in 10 fractions over 2 weeks at both institutions. After WBRT, a follow-up neuroradiological examination was performed with contrast-enhanced x-ray computed tomography (CT) or magnetic resonance imaging (MRI) every 2–6 months. Written consent was obtained from all patients or their relative before WBRT. This study was approved by the Institutional Review Board of Niigata University Hospital (Study #1449).

### 4.2. Neurocognitive Function Assessment

Each patient’s NCF was assessed with the HVLT-R (Japanese version), consisting of the total recall (TR), delayed recall (DR), and delayed recognition (DRecog) subdomains. The HVLT-R was performed before (baseline) and at 4 months and 8 months after the completion of WBRT. The HVLT-*R* test battery contains six different question forms so that the participant cannot memorize the answers. The interval between the day exactly 4 or 8 months after WBRT and the actual date of the follow-up HVLT-R was <1 month. The HVLT-R scores were analyzed in two different ways. One way was an inter-group or intra-group (the baseline, 4, and 8 months) comparison of raw scores with nonparametric tests, and the other way was to judge whether the 4- or 8-month score of a participant was significantly declined compared to the baseline beyond a specific cutoff value. Significant deterioration in an individual’s NCF was defined as a drop from the baseline score by ≥5 points for TR, ≥3 points for DR, and ≥2 points for DRecog [10]. For example, if the baseline TR score was 15 and the 8-month TR score was 9, the 8-month TR score was judged as declined compared to the baseline.

### 4.3. Statistical Analyses

The association between the patient characteristics and the baseline HVLT-R scores in each subdomain was evaluated with the Mann-Whitney *U*-test and the Kruskal-Wallis test. Among the patients who underwent the 4-month examination and did not undergo the 8-month examination, the HVLT-R scores at the baseline and 4 months after WBRT were compared using the Wilcoxon signed rank test. Among the patients who completed the 8-month examination, we used the Friedman test to compare the baseline, 4-month, and 8-month scores. A univariate Fisher’s exact test was performed to determine the factors associated with a significant decline from the baseline score in each HVLT-R subdomain. A *p*-value <0.05 was considered significant.

All statistical analyses were performed with EZR (Saitama Medical Center, Jichi Medical University, Saitama, Japan), which is a graphical user interface for R (The R Foundation for Statistical Computing, Vienna, Austria) [13].

## 5. Conclusions

The results of this study demonstrated the feasibility of the HVLT-R Japanese version for evaluating the patients’ NCF after brain irradiation. We found that the patients with either older age or lower KPS had impaired NCF as assessed with the HVLT-R at the baseline. The four-month NCF deterioration was associated with both the KPS and poor outcome. The WBRT dose fractionation did not have an impact on the incidence of the decline in the NCF at four or eight months. The early decline in the NCF is characteristic of the worse-prognosis group, and thus the NCF of favorable-prognosis patients might better be evaluated later (i.e., at six months or later) after irradiation.

## Figures and Tables

**Table 1:** Patient characteristics of each WBRT dose group.

Variable	WBRT Dose Group			*p*
	25 Gy (*n* = 11)	30 Gy (*n* = 18)	35 Gy (*n* = 16)	
Age: median (range)	65 (52–77)	65 (45–86)	66 (39–81)	0.95
Cranial lesion (*n*):				
BM	0	7	16	<0.001
Dissemination	0	6	0	
NHL	0	3	0	
Skull metastasis	0	2	0	
None	11	0	0	
Gender (*n*):				
Female	3	10	11	0.10
Male	8	8	5	
KPS (*n*):				
≤70	0	7	5	0.062
80–100	11	11	11	
Primary site (*n*):				
Breast	0	4	3	<0.001
NSCLC	0	10	8	
SCLC	11	1	1	
NHL	0	3	0	
others	0	0	4	
Total number of examinations (*n*):				
Two (baseline and 4 mos)	1	8	7	0.11
Three (baseline, 4, and 8 mos)	10	10	9	
Reason for discontinuation (*n*):				
Denial	0	2	0	0.52
Death from cancer	1	3	4	
Worsened general condition	0	3	3	

WBRT: Whole-brain radiation therapy, BM: Brain metastasis, NHL: Non-Hodgkin lymphoma, KPS: Karnofsky Performance Status, NSCLC: Non-small cell lung cancer, SCLC: Small-cell lung cancer.

**Table 2:** Age, KPS, leptomeningeal dissemination and total number of examinations.

Variable	Total Number of Examinations		*p*
	2 (baseline and 4 mos) (*n* = 16)	3 (baseline, 4, and 8 mos) (*n* = 29)	
Age:			
≤65	8 (32%)	17 (68%)	0.76
≥66	8 (40%)	12 (60%)	
KPS:			
≤70	8 (67%)	4 (33%)	0.014
80–100	8 (24%)	25 (76%)	
Dissemination			
No	15 (38%)	24 (62%)	0.40
Yes	1 (17%)	5 (83%)	

KPS: Karnofsky Performance Status.

**Table 3:** Baseline HVLT-R scores and associated clinical factors.

Subdomain	Variable	*n*	Average (SD)	*p*
TR	KPS:			
	≤70	12	13.1 (6.4)	0.0053
	80–100	33	19.9 (6.3)	
	Age:			
	≤65	25	20.0 (6.2)	0.030
	≥66	20	15.7 (7.3)	
	Dose (Gy):			
	25	11	17.0 (4.6)	0.19
	30	18	16.2 (8.1)	
	35	16	20.9 (6.4)	
	Cranial Lesion:			
	BM	23	18.1 (7.0)	0.011
	Dissemination	6	23.5 (4.6)	
	NHL	3	6.7 (5.9)	
	Skull metastasis	2	24.5 (2.1)	
	None	11	17.0 (4.6)	
DR	KPS:			
	≤70	12	3.0 (2.8)	0.012
	80–100	33	6.1 (3.4)	
	Age:			
	≤65	25	6.0 (3.3)	0.080
	≥66	20	4.3 (3.6)	
	Dose (Gy):			
	25	11	5.4 (2.2)	0.21
	30	18	4.2 (4.0)	
	35	16	6.4 (3.5)	
	Cranial Lesion:			
	BM	23	4.9 (3.8)	0.014
	Dissemination	6	8.5 (2.2)	
	NHL	3	0.0 (0.0)	
	Skull metastasis	2	6.5 (2.1)	
	None	11	5.4 (2.2)	
DRecog	KPS:			
	≤70	12	7.5 (3.4)	0.0078
	80–100	33	10.3 (1.7)	
	Age:			
	≤65	25	10.4 (1.6)	0.031
	≥66	20	8.5 (3.1)	
	Dose (Gy):			
	25	11	9.8 (1.7)	0.27
	30	18	8.6 (3.4)	
	35	16	10.4 (1.6)	
	Cranial Lesion:			
	BM	23	9.7 (2.1)	0.021
	Dissemination	6	11.2 (1.0)	
	NHL	3	3.3 (2.9)	
	Skull metastasis	2	11.0 (1.4)	
	None	11	9.8 (1.7)	

The *p*-value was Bonferroni-adjusted in the comparison among different WBRT dose groups or cranial lesions.

**Table 4:** HVLT-R scores of the patients who underwent three examinations (baseline, four, and eight months) by WBRT dose fractionation.

Dose (Gy)	*n*	Subdomain	Baseline Score Avg. (SD)	4-Month Score Avg. (SD)	8-Month Score Avg. (SD)	*p* (Friedman Test)
25	10	TR	16.7 (4.8)	19.9 (3.7)	18.4 (4.7)	0.078
		DR	5.4 (2.3)	6.1 (2.1)	6.8 (2.7)	0.19
		DRecog	9.6 (1.7)	9.0 (2.2)	9.9 (1.6)	0.30
30	10	TR	18.9 (9.1)	21.1 (6.9)	21.5 (4.2)	0.71
		DR	5.7 (4.4)	6.9 (3.8)	7.1 (3.7)	0.44
		DRecog	9.4 (3.9)	9.4 (2.6)	10.3 (2.4)	0.23
35	9	TR	20.3 (6.8)	19.6 (7.5)	22.3 (7.6)	0.52
		DR	6.2 (4.2)	5.9 (3.4)	6.4 (4.0)	0.88
		DRecog	10.3 (1.5)	9.4 (2.3)	9.4 (3.1)	0.96

**Table 5:** Significant decline in four-month individual HVLT-R scores and associated factors.

Subdomain	Variable	Declined, *n* (%)	Not Declined, *n* (%)	*p*
TR	Age:			
	≤65	4 (16)	21 (84)	0.30
	≥66	6 (30)	14 (70)	
	Dose (Gy):			
	25	1 (0.9)	10 (90.9)	0.22
	30	3 (17)	15 (83)	
	35	6 (37)	10 (63)	
	KPS:			
	≤70	6 (50)	6 (50)	0.013
	80–100	4 (12)	29 (88)	
	Total number of examinations:			
	Two (baseline and 4 mos)	8 (50)	8 (50)	0.0017
	Three (baseline, 4, and 8 mos)	2 (7)	27 (93)	
DR	Age:			
	≤65	6 (24)	19 (76)	1
	≥66	5 (25)	15 (75)	
	Dose (Gy):			
	25	2 (18)	9 (82)	0.75
	30	4 (22)	14 (78)	
	35	5 (31)	11 (69)	
	KPS:			
	≤70	4 (33)	8 (67)	0.45
	80–100	7 (21)	26 (79)	
	Total number of examinations:			
	Two (baseline and 4 mos)	7 (44)	9 (56)	0.035
	Three (baseline, 4, and 8 mos)	4 (14)	25 (86)	
DRecog	Age:			
	≤65	8 (32)	17 (68)	1
	≥66	7 (35)	13 (65)	
	Dose (Gy):			
	25	4 (36)	7 (64)	0.41
	30	4 (22)	14 (78)	
	35	7 (44)	9 (56)	
	KPS:			
	≤70	5 (42)	7 (58)	0.50
	80–100	10 (33)	23 (67)	
	Total number of examinations:			
	Two (baseline and 4 mos)	7 (44)	9 (56)	0.33
	Three (baseline, 4, and 8 mos)	8 (28)	21 (72)	

**Table 6:** Significant decline in eight-month individual HVLT-R scores and associated factors.

Subdomain	Variable	Declined, *n* (%)	Not Declined, *n* (%)	*p*
TR	Age:			
	≤65	2 (12)	15 (88)	1
	≥66	1 (8)	11 (92)	
	Dose (Gy):			
	25	1 (10)	9 (90)	1
	30	1 (10)	9 (90)	
	35	1 (11)	8 (89)	
	KPS:			
	≤70	0 (0)	4 (100)	1
	80–100	3 (12)	22 (88)	
DR	Age:			
	≤65	0 (0)	17 (100)	0.060
	≥66	3 (25)	9 (75)	
	Dose (Gy):			
	25	1 (10)	9 (90)	1
	30	1 (10)	9 (90)	
	35	1 (11)	8 (89)	
	KPS:			
	≤70	1 (25)	3 (75)	0.37
	80–100	2 (8)	23 (92)	
DRecog	Age:			
	≤65	2 (12)	15 (88)	0.20
	≥66	4 (33)	8 (67)	
	Dose (Gy):			
	25	1 (10)	9 (90)	0.46
	30	2 (20)	8 (80)	
	35	3 (33)	6 (67)	
	KPS:			
	≤70	2 (50)	2 (50)	0.18
	80–100	4 (16)	21 (84)	

## Abbreviations

BM: brain metastasis
CNS: central nervous system
COWAT: Controlled Oral Word Association Test
DR: delayed recall
DRecog: delayed recognition
HVLT: Hopkins Verbal Learning Test
HVLT-R: Hopkins Verbal Learning Test-Revised
KPS: Karnofsky Performance Status
LD-SCLC: limited-disease small cell lung cancer
MMSE: Mini-Mental State Examination
NCF: neurocognitive function
NHL: non-Hodgkin lymphoma
NSCLC: non-small cell lung cancer
RTOG: Radiation Therapy Oncology Group
SCLC: small-cell lung cancer
SRS: stereotactic radiosurgery
TMT: Trail Making Test
TR: total recall
WBRT: whole-brain radiation therapy
